# Investigating sources of non-response bias in a population-based seroprevalence study of vaccine-preventable diseases in the Netherlands

**DOI:** 10.1186/s12879-024-09095-5

**Published:** 2024-02-23

**Authors:** Abigail Postema, José A. Ferreira, Fiona van der Klis, Hester de Melker, Liesbeth Mollema

**Affiliations:** 1 Centre for Infectious Disease Control, National Institute for Public Health and the Environment, Bilthoven, The Netherlands; 2 Public Health and Health Services, National Institute for Public Health and the Environment, Bilthoven, The Netherlands

**Keywords:** Seroprevalence, National immunization programme, Survey, Non-response bias

## Abstract

**Background:**

PIENTER 3 (P3), conducted in 2016/17, is the most recent of three nationwide serological surveys in the Netherlands. The surveys aim to monitor the effects of the National Immunisation Programme (NIP) by assessing population seroprevalence of included vaccine preventable diseases (VPDs). The response rate to the main sample was 15.7% (*n* = 4,983), following a decreasing trend in response compared to the previous two PIENTER studies (P1, 55.0%; 1995/1996 [*n* = 8,356] and P2, 33.0%; 2006/2007 [*n* = 5,834]). Non-responders to the main P3 survey were followed-up to complete a “non-response” questionnaire, an abridged 9-question version of the main survey covering demographics, health, and vaccination status. We assess P3 representativeness and potential sources of non-response bias, and trends in decreasing participation rates across all PIENTER studies.

**Methods:**

P3 invitees were classified into survey response types: Full Participants (FP), Questionnaire Only (QO), Non-Response Questionnaire (NRQ) and Absolute Non-Responders (ANR). FP demographic and health indicator data were compared with Dutch national statistics, and then the response types were compared to each other. Random forest algorithms were used to predict response type. Finally, FPs from all three PIENTERs were compared to investigate the profile of survey participants through time.

**Results:**

P3 FPs were in general healthier, younger and higher educated than the Dutch population. Random forest was not able to differentiate between FPs and ANRs, but when predicting FPs from NRQs we found evidence of healthy-responder bias. Participants of the three PIENTERs were found to be similar and are therefore comparable through time, but in line with national trends we found P3 participants were less inclined to vaccinate than previous cohorts.

**Discussion:**

The PIENTER biobank is a powerful tool to monitor population-level protection against VPDs across 30 years in The Netherlands. However, future PIENTER studies should continue to focus on improving recruitment from under-represented groups, potentially by considering alternative and mixed survey modes to improve both overall and subgroup-specific response. Whilst non-responder bias is unlikely to affect seroprevalence estimates of high-coverage vaccines, the primary aim of the PIENTER biobank, other studies with varied vaccination/disease exposures should consider the influence of bias carefully.

**Supplementary Information:**

The online version contains supplementary material available at 10.1186/s12879-024-09095-5.

## Introduction

Sero-epidemiological surveys are powerful tools for infectious disease surveillance. They allow the direct measurement of exposure to an infectious agent, or to a vaccination, across a population [1, 2]. In the Netherlands, national serosurveys have been conducted every 10 years since 1996 to monitor the success of the National Immunisation Programme (NIP). These are known as the PIENTER studies.

These successive studies offer a unique insight into the serostatus of the Dutch population and have provided evidence to support recommendations on vaccination policies in the Netherlands [3–5]. For example, through monitoring the seroprevalence of protective antibodies against measles, mumps and rubella, groups at risk of future infections due to waned immunity or lower vaccination uptake were able to be identified [3]. Further, the evaluation of tetanus antitoxin seroprevalence has indicated hat the Dutch population is highly protected against tetanus under existing routine vaccination policy, with only 10% of those eligible for post-exposure prophylaxis found to be insufficiently protected [4]. The PIENTER serosurveys have also supported evaluation of newly implemented vaccines, when estimating the seroprevalence of serogroup C Neisseria meningitis antibodies prior to, and following, the introduction of meningococcal c vaccinations [5]. It is therefore important that estimates generated from these surveys be reliable and generalizable to the Dutch population.

Survey non-response is one of the largest challenges to survey research, with decreasing survey response rates experienced globally, irrespective of country or survey topic [6]. Much time and effort has been invested by the survey research community into developing methods to explain, and thereby prevent or adjust for, non-response [7–9]. As with many European health surveys, the PIENTER studies have experienced decreasing response rates at each iteration [10]. The most recent, PIENTER 3 (P3), was conducted in 2016/17, and comprised of a main sample, henceforth known as the National Sample, and oversamples of under-represented groups and groups of special interest. PIENTER 2 (P2) and PIENTER 1 (P1) were conducted 10 and 20 years prior to this, with defined oversampling groups differing between PIENTER iterations. However, the target population and sampling approach for the National Sample remained unchanged. For the National Sample, P3, P2 and P1 had response rates of 15.7%, 33%, and 55%, respectively [11–13].

Low response rates can severely impact not only the representativeness of a sample, but also the reliability of any estimates made, due to the introduction of non-response biases [14]. However, a survey response rate alone is not a robust indicator of the presence of non-response bias [15–17]. Rather, the impact of non-response bias on survey-derived estimates depends upon how participation behaviours are associated with the nature of the survey questions [18, 19].

This paper aims to describe the representativeness of the P3 sample and investigate potential sources of non-response bias. First, we describe the participants of P3, and compare them to the Dutch general population. Secondly, we investigate potential determinants of non-response bias by comparing demographic- and survey-derived characteristics of responders and non-responders. We then aim to identify factors, and combinations of them, which explain participation in the survey. Lastly, we compare the participants from all three PIENTERs to determine whether the characteristics of PIENTER participants have changed through time.

The P3 data and samples will be used for a large variety of health studies. Subsequently, the influence of potential non-response bias must be considered on a case-by-case basis. We hope that the findings of this paper will support future researchers and policy makers when interpreting and applying P3 findings to the broader Dutch population. Further, we hope that our findings may inform strategies to improve response to future health surveys, PIENTER or otherwise.

## Methods

### PIENTER 3 study population and sample design

#### Description of PIENTER3 (P3) sampling for the national sample

As in P1 and P2, a two-stage sample was drawn from the Dutch national population [11, 12]. Eight municipalities were sampled, with probabilities proportional to their population sizes, from five geographic regions of a similar population size. An age-stratified sample was then drawn from the register of each municipality, henceforth referred to as the National Sample. Sampling and recruitment strategies for P3 have been described in further detail elsewhere [13].

#### Oversampling of non-western migrants (NWMs)

An additional oversample of non-western migrants (NWMs) was drawn due to the low response rates seen in P1 and P2 for this subgroup [11, 12]. A stratified sample of NWMs residing in 9 of the 40 municipalities and not included in the National Sample (sampled without replacement) were invited to participate.

#### Data collection and recruitment methods

Invitees were contacted by post and asked to complete an online or paper questionnaire. The invitation contained an appointment at the study clinic to have samples taken but indicated that “walk-in” appointments were possible. Before the scheduled appointment invitees received a reminder letter and telephone call. At the appointment informed consent was obtained and biological samples were taken. Each participant received a €25 incentivisation payment and was offered a further €25 for the donation of additional biological materials.

Invitees were contacted again if they did not attend their appointment. Invitees that did not want to attend clinic but wished to participate were sent a self-collection kit, to donate a finger-prick dried blood spot (DBS) sample. Invitees that indicated that they did not want to participate were followed up to ascertain why they did not want to participate and to complete a highly shortened version of the main questionnaire. Herein referred to as a “Non-Response Questionnaire”, this short follow-up survey was conducted by an external study call-centre. It comprised in total 9 questions, which are presented in Additional File 1. Included questions were identical to those in the main questionnaire, aside from the open question asking for reasons for non-participation.

### PIENTER 3 Survey participation behaviour– response types

#### Defining response types

Based on participation behaviour all invitees were assigned a Response Type:

**Full participants (FPs)** submitted a questionnaire and donated at least one biological sample at a study clinic. Biological samples included blood, saliva, oro/nasopharyngeal swabs, and faeces.

**Questionnaire-only participants (QOs)** submitted a questionnaire but did not attend a study clinic to submit any biological samples. Participants with a DBS were classed as QOs as they did not physically attend a clinic.

**Non-responders who submitted a Non-Response Questionnaire (NRQs)** did not submit a questionnaire or attend clinic but completed a telephone NRQ.

**Absolute non-responders (ANRs)** did not attend a study clinic and did not submit a non-response questionnaire.

#### Response rates

Using the above response types, the survey response rate is calculated as Response Rate 1 of the American Association for Public Opinion Research (AAPOR) 2016 standard definitions [20]:

$$AAPOR\,Response\,Rate1 = \frac{{FP}}{{\left( {{\text{FP}} + {\text{QO}}} \right) + \left( {{\text{NRQ}} + {\text{ANR}} + {\text{O}}} \right)}}$$

Some participants were excluded due to missing questionnaire data, despite having provided a biological sample, and were classed as ‘Other’ (O).

#### Comparison of response type characteristics

To investigate sources of non-response bias, demographic characteristics were compared between the four response types, presenting counts and proportions for categorical variables.

Chi-squared tests were used to test for at least one difference and for pairwise differences between response types with respect to each variable. Variance across the four response types was assessed using chi-square tests adjusted using the Benjamini-Hochberg multiple testing procedure, at a false discovery rate of 0.05 [21].

### Representativeness of PIENTER 3 sample

To investigate representativeness, we compared basic demographic and questionnaire-derived characteristics of all P3s FPs to those of the Dutch population. Population data were correct as of the 1^st of^ January 2016, (CBS Nederland).

### Predicting response types using random forests (RFs)

Random forest (RF) is a non-parametric prediction algorithm. It is constructed with a large data set consisting of outcomes of individuals, such as vaccination status or as in this present study response type, coded in binary (1 vs. 0), and the concomitant values of many predictor variables (such as age, sex, education, etc.) [22, 23].

A RF consists of a collection of decision trees. A decision tree is a schematic recipe for deciding which outcome is more likely for an individual on the basis of the particular combination of the individual’s predictor variables. Each tree is constructed with a random subsample of the whole data set in such way that all trees are somewhat different. Although each individual decision tree can deliver a prediction of the outcome, and individual decision tree predictions can be rather erratic, the overall prediction is based on the “forest” that they make up and is more accurate. In the case of a binary outcome, the overall prediction is the ‘majority vote’ of the trees, namely which outcome, 0 or 1, was most commonly predicted amongst the individual decision trees forming the forest.

As with any other statistical prediction, RFs are assessed in terms of the accuracy in predicting outcomes. When generating the predictions, the observed outcome is removed from the dataset. The final predicted outcome is then compared to the actual observed outcome. In doing so, RF can assess the accuracy of those predictions. With binary outcomes, the most concrete measures of (in)accuracy include the probability of misclassification (pmc, the probability of predicting an individual’s outcome incorrectly), the sensitivity (the probability of predicting an individual’s outcome correctly when the individual’s outcome is ‘positive’ or 1) and the specificity (the probability of predicting the outcome correctly when the individual’s outcome is ‘negative’ or 0). The RF algorithm provides reliable estimates of these three quantities [23]. In addition, it provides measures of variable importance, which play a crucial role in the present study.

RF measures the importance of a variable in predicting an outcome by computing the percent increase in pmc that results from a random permutation of the values of that variable in the data set [22, 23]. If the permuted value of a variable tends to worsen the prediction, that variable is regarded as important in determining the outcome. If, overall, the prediction is about as accurate with the permuted and correct inputs, the variable is regarded as unimportant [22, 23]. Confusion matrices were constructed to indicate the sensitivity and specificity of the prediction, and variable importance plots were created to visualise which variables best predicted response type separation.

In our analyses, we used RF to predict response type as a nominal outcome using demographic and questionnaire-derived variables as predictors. To account for potential variations in outcome across geographical regions, we also used participant coordinates based on their residential postcode (PC4).

We conducted three analyses: comparing FPs to ANRs, FPs to NRQs, and FPs to QOs. In each comparison, FP was the “positive” outcome, and its complement response type was the “negative” outcome. Variable definitions are provided in Additional File 2.

### Comparison of participants from PIENTER 1, PIENTER 2, and PIENTER 3

Using a combination of demographic and questionnaire-derived variables, RF predicted which PIENTER year each participant originated from. Participants aged over 79 were excluded from P3, as P1 and P2 did not sample this age group. The NWM oversamples of P3 and P2 were also excluded, as oversampling was not conducted during P1.

In order to obtain estimates of sensitivity and specificity from the RF analyses, the NS samples were analysed in pairs; P1 with P2, P2 with P3 and P1 with P3. Data regarding the degree of urbanisation were excluded when comparing P1 to P2 and P2 to P3, as P2 data on urbanisation were limited. Variable definitions can be found in Additional File 2.

### Analytical considerations

As is common in survey datasets, we were faced with the challenge of missing data. Based on *a priori* knowledge of survey response behaviours and on differences seen in key sociodemographic measures across the P3 response types, we expect that the missing data of the variables included in our analyses could not be considered Missing Completely at Random (MCAR) or Missing at Random (MAR). The degree of missing data present in variables common to different response types varied, with missingness increasing for participants with less survey engagement. Consequently, missingness in itself must be somewhat informative, indicating potential underlying differences between the response type groups, or at least a gradient in the willingness of response type groups to divulge certain information.

Where missing data occurred in a categorical variable, they were assigned a category level of its own and included in all analyses, unless otherwise stated. There were no missing data in the continuous variables.

All analyses were conducted in R v 4.0.3 (RStudio Server 1.4.1103), using the “stats” (version 3.6.0) and “randomForest” (version 4.6–14) packages [23–25].

## Results

### PIENTER 3 study population and response rates

In total 40,065 individuals were invited from 40 municipalities. 167 individuals were excluded due to non-delivery or inability to participate for medical reasons. From a net sample of 39,898, a total of 31,714 individuals were invited in the National Sample and 8,184 were invited in the Non-Western Migrant (NWM) oversample.

The overall response rate was 13.9% (National Sample + NWM; 5,553 / 39,898). For the National Sample alone, the response rate was 15.7% (4,983 / 31,714), and for the NWM oversample the response rate was 7.0% (570 / 8,184). Response rates varied by age, gender, and urbanisation, as well as migration background. A detailed description of the PIENTER 3 response rates, overall and by subgroups, is provided in Verberk et al. 2019. However, for convenience a brief overview of response rates by these subgroups is presented in Additional File 3.

### PIENTER 3 Survey participation behaviour - response types

**Fig. 1:**
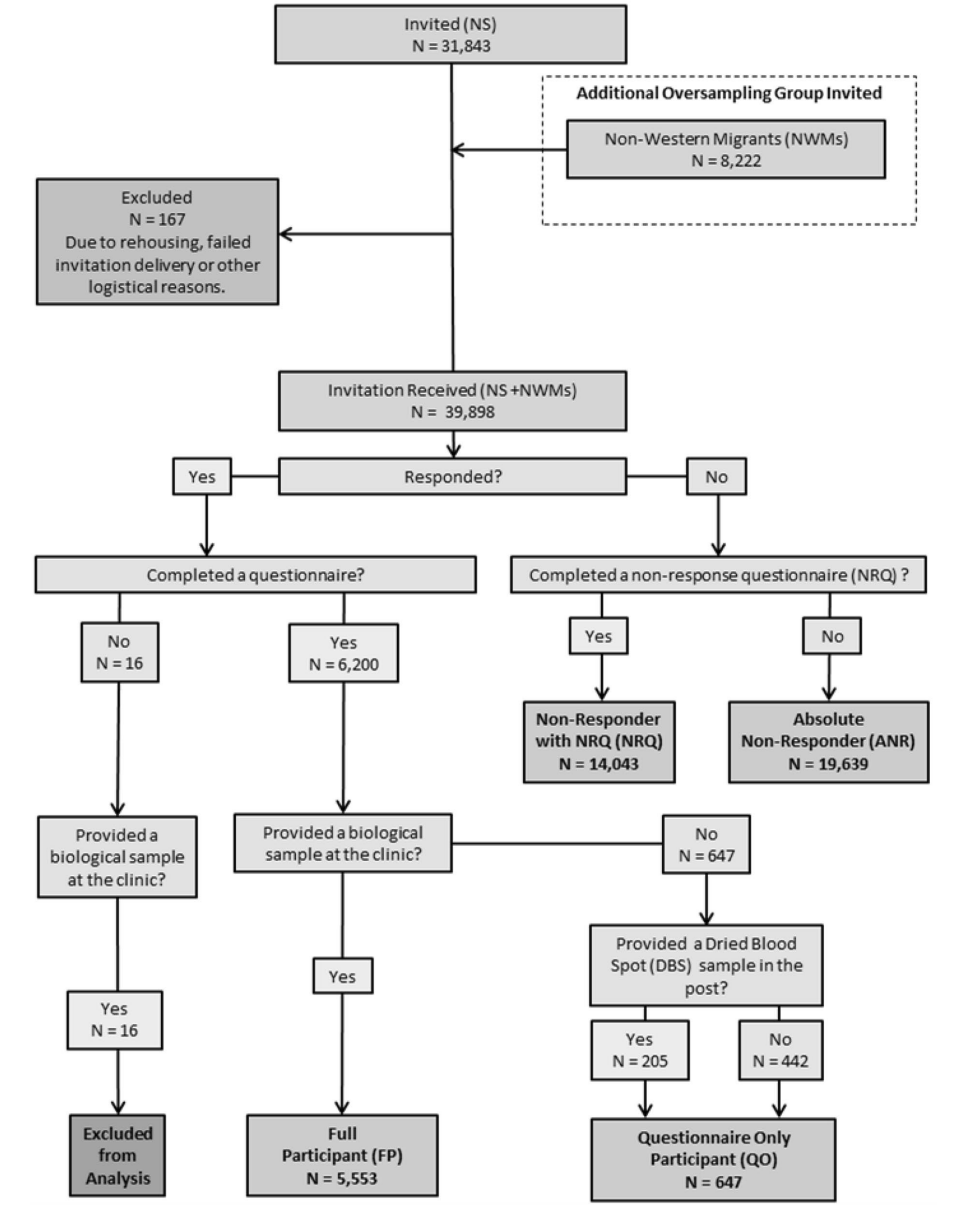
Flowchart of response type allocation based on participation behaviour for PIENTER 3

Considering both samples together (NS + NWM), 5,553 were classified as FPs, 647 as QOs, 14,043 as NRQs and 19,639 as ANRs (Fig. 1). A further 16 participants were excluded due to missing questionnaire data, despite having a biological sample, and are labelled as ‘Other’ (O).

When comparing characteristics between response types, we found that the variance in distributions across the four response types was significant regarding all tested characteristics (Table 1).

**Table 1:** Demographic characteristics of P3 participants in each response type* category *Response Types - FP; Full Participant, QO; questionnaire only participant, NRQ; non-response questionnaire participant, ANR; absolute non-responder

Variable	FPs *N* = 5,553 (13.9%)	QOs *N* = 647 (1.6%)	NRQs *N* = 14,043 (35.2%)	ANRs *N* = 19,639 (49.2%)	*p*-value (Chi^2^ with BH adjustment)
Age-group (in years)					
< 10	1051 (18.9)	131 (20.3)	3263 (23.2)	5535 (28.2)	
10–19	608 (11.0)	70 (10.8)	1201 (8.6)	1363 (6.9)	
20–29	766 (13.8)	129 (20.0)	2335 (16.6)	3777 (19.2)	
30–39	697 (12.6)	102 (15.8)	1755 (12.5)	2974 (15.1)	
40–49	634 (11.4)	77 (11.9)	1771 (12.6)	1858 (9.5)	
50–64	938 (16.9)	78 (12.1)	1945 (13.9)	2076 (10.6)	
65–79	785 (14.1)	43 (6.7)	1452 (10.3)	1618 (8.2)	
80+	74 (1.3)	17 (2.6)	321 (2.3)	437 (2.2)	< 0.001
Sex					
Male	2538 (45.7)	314 (48.5)	8052 (57.3)	10,704 (54.5)	
Female	3015 (54.3)	333 (51.5)	5991 (42.7)	8935 (45.5)	< 0.001
Degree of urbanisation					
Very high	1167 (21.0)	200 (30.9)	3966 (28.2)	7509 (38.2)	
High	1816 (32.7)	207 (32.0)	4122 (29.4)	6020 (30.7)	
Middle	1064 (19.2)	110 (17.0)	2362 (16.8)	3161 (16.1)	
Low	1018 (18.3)	94 (14.5)	2164 (15.4)	1992 (10.1)	
Very low	488 (8.8)	36 (5.6)	1429 (10.2)	956 (4.9)	< 0.001
Migration Background					
Dutch	4352 (78.4)	464 (71.7)	9150 (65.2)	10,436 (53.1)	
Other Western	366 (6.6)	49 (7.6)	1100 (7.8)	1784 (9.1)	
Moroccan or Turkish	134 (2.4)	27 (4.2)	1223 (8.7)	2678 (13.6)	
Antillean, Surinamese or Aruban	269 (4.8)	53 (8.2)	1247 (8.9)	2087 (10.6)	
Other Non-Western	431 (7.8)	54 (8.4)	1320 (9.4)	2634 (13.4)	< 0.001
Missing	1 (0.02)	0 (0.0)	3 (0.02)	19 (0.1)	
SES					
Lowest	1625 (29.3)	220 (34.0)	5034 (35.8)	7780 (39.6)	
Low	885 (15.9)	97 (15.0)	2336 (16.6)	3242 (16.5)	
Middle	983 (17.7)	102 (15.8)	2267 (16.1)	2518 (12.8)	
High	1182 (21.3)	132 (20.4)	2452 (17.5)	3201 (16.3)	
Highest	873 (15.7)	95 (14.7)	1934 (13.8)	2886 (14.7)	< 0.001
Missing	5 (0.09)	1 (0.2)	20 (0.1)	11 (0.06)	

*Response Types - FP; Full Participant, QO; questionnaire only participant, NRQ; non-response questionnaire participant, ANR; absolute non-responder.

### Representativeness of the PIENTER 3 sample - full participants (FPs) only

Compared to national figures reported by CBS, FPs had a higher proportion of females and under 20-year-olds, and a lower proportion of older adults aged 40–69 years. FPs from areas of very high or very low degrees of urbanisation were underrepresented. FPs contained a lower proportion of participants with either Moroccan, Turkish or Western migration backgrounds, but had a larger proportion of participants with Surinamese, Antillean or other non-western migration backgrounds. FPs were more highly educated, had a higher household income and reported themselves to be healthier than the general Dutch population. A detailed breakdown is presented in Additional File 4.

### Predicting response type

#### Full participant (FP) or absolute non-responder (ANR)

**Fig. 2:**
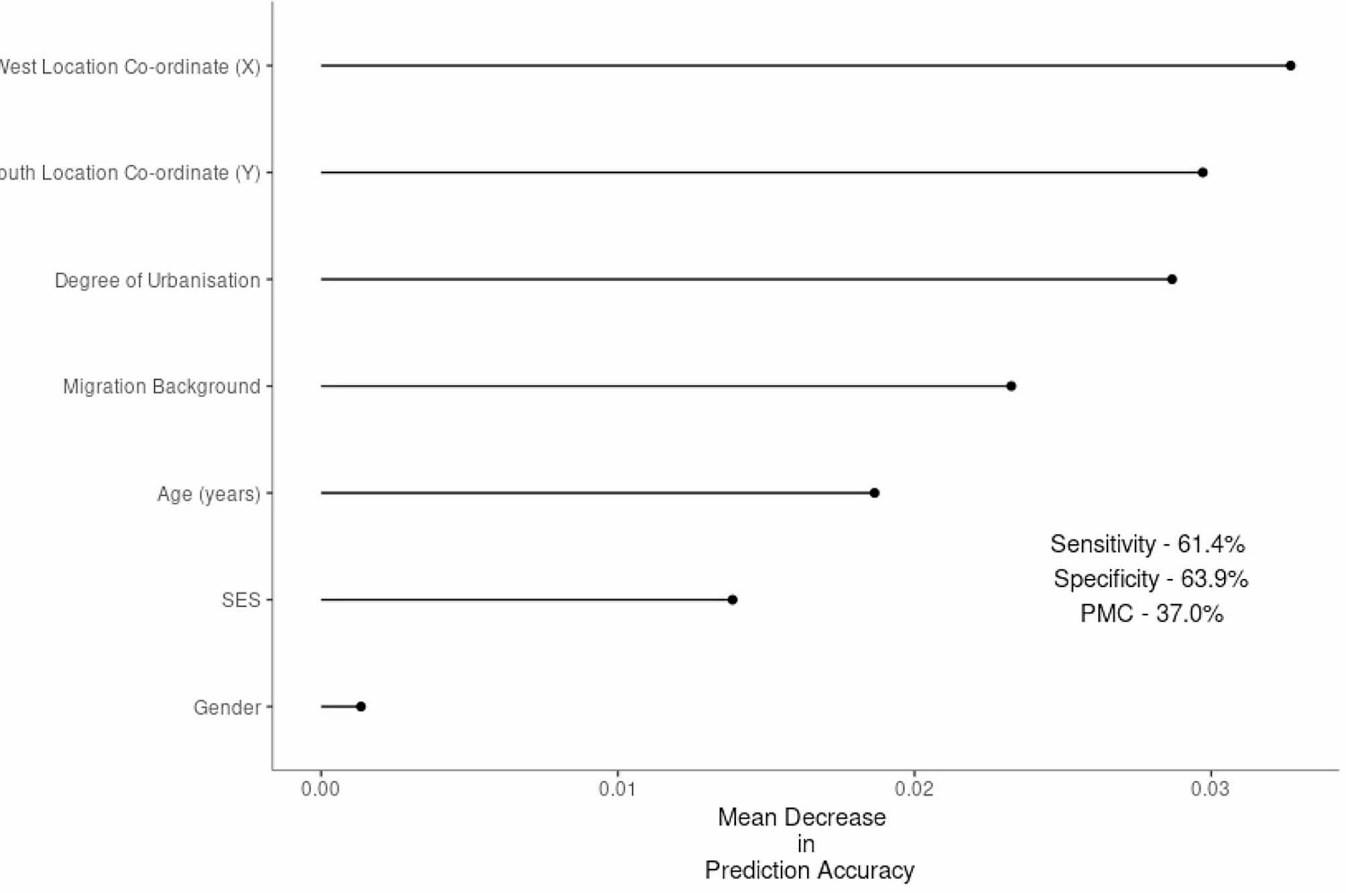
Ordered variable importance for predicting an Absolute Non-Response from a Full Participant in P3

When predicting response type among ANRs and FPs, the most important predictors were the degree of urbanisation and geographical location (X and Y co-ordinates of districts within municipalities) of the participant, closely followed by migration background (Fig. 2.). The model had a probability of misclassification (pmc) of 37.0%, a sensitivity of 61.4%, a specificity of 63.9% and an accuracy (1– pmc) of 63.0% (Table 2). The majority of ANRs originated from very highly urbanised areas, with FPs more likely to originate from highly or moderately urbanised areas (Table 1). Less than 25% of FPs were of a non-Dutch migration background, compared to 50% of ANRs (Table 1).

**Table 2:** Performance metrics of each random forest model by response type comparison. PMC is the probability of misclassification by the model, and accuracy is 1– PMC.

Response Type Model	Number of each Response Type (*n*: *n*)	Sensitivity (%)	Specificity (%)	PMC (%)	Accuracy (%)
***ANR vs. FP***	19,639: 5,553	61.4	63.9	37.0	63.0
***NRQ vs. FP***	14,043: 5,553	58.9	91.5	18.0	82.0
***NRQ vs. FP (complete case)***	9,451: 5,546	59.4	86.6	23.3	76.7
***QO vs. FP***	647: 5,553	99.4	10.7	9.8	90.2

#### Full participant (FP) or non-response questionnaire (NRQ) participant

**Fig. 3:**
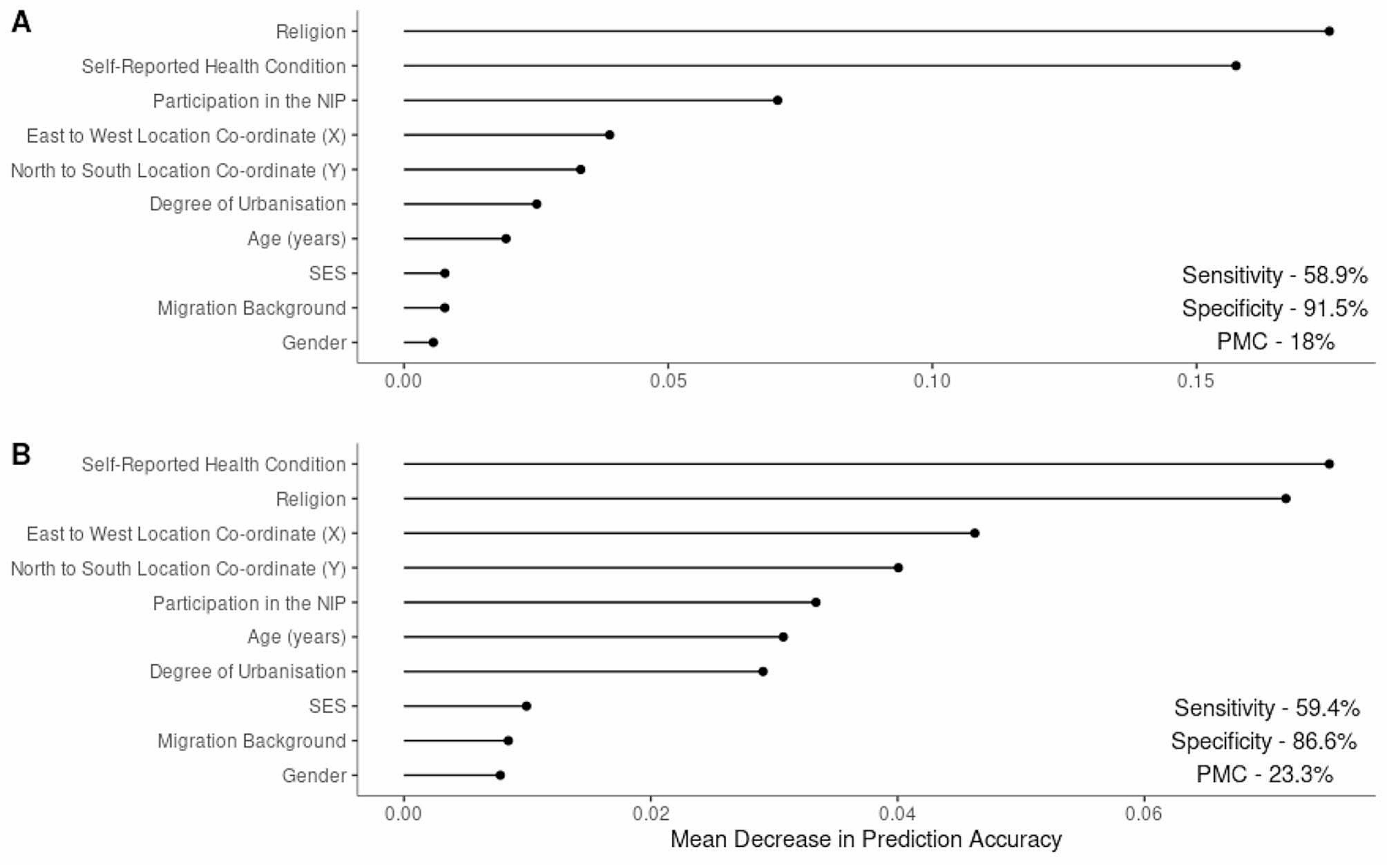
Panel **A**. Ordered variable importance for predicting an NRQ from a FP using all data, in P3 (ANR *n* = 14,043. FP *n* = 5,553). Panel **B**– As panel **A**, but excluding participants who had missing data for all three of the NRQ-derived variables “Religion,” “Self-Reported Health Condition,” and “NIP Participation” (ANR n complete data = 9,451, FP n complete data = 5,546)

When predicting response type among NRQs and FPs the most important predictors were religion and self-reported health condition, followed by NIP participation (Fig. 3 Panel A). The model had a pmc of 18.0%, a sensitivity of 58.9%, a specificity of 91.5% and an accuracy (1– pmc) of 82.0% (Table 2).

However, there was a considerable amount of missing data for NRQs in these three variables, with 32.7% (4,592/14,043) of all NRQs missing data across all three additional predictor variables. Naturally, the refusal to provide data on such topics as religion is itself informative. However, to check whether missingness on a variable was overly influencing the random forest variable importance rankings, we re-ran the RF excluding any participants who had missing data across all three NRQ derived variables. Whilst the PMC increased in the second analysis, the variable importance order remained relatively similar (Fig. 3). The most important variables in this analysis were self-reported health condition, religion, and geographical location (X and Y co-ordinates) of the participant (Fig. 3 Panel B). The “complete case” model had a pmc of 23.3%, a sensitivity of 59.4%, a specificity of 86.6% and an accuracy (1– pmc) of 76.6% (Table 2).

While proportions of those reporting poor or very poor health satisfaction were similar for NRQs and FPs, 25.9% of FPs reported to have very good health, compared to only 7.5% of NRQs. 76.9% of NRQs reported most frequently to have good health satisfaction, compared to 58.6% of FPs. The proportion of those reporting to hold any religious belief was higher in FPs than NRQs, with more NRQs reporting to have no religious belief compared to FPs. Following exclusion of NRQs with entirely missing data, the proportion of missing values for health satisfaction and religion was 8.1% and 11.0% for NRQs respectively, and 1.3% and 6.9% for FPs, respectively. Based on this, we conclude that the categorical level for missingness increased the accuracy of the prediction but did not necessarily influence the order of the variables of importance. The distribution of NRQ and FP questionnaire characteristics are provided in Additional File 5.

#### Full participant (FP) or Questionnaire only (QO) participant

**Fig. 4:**
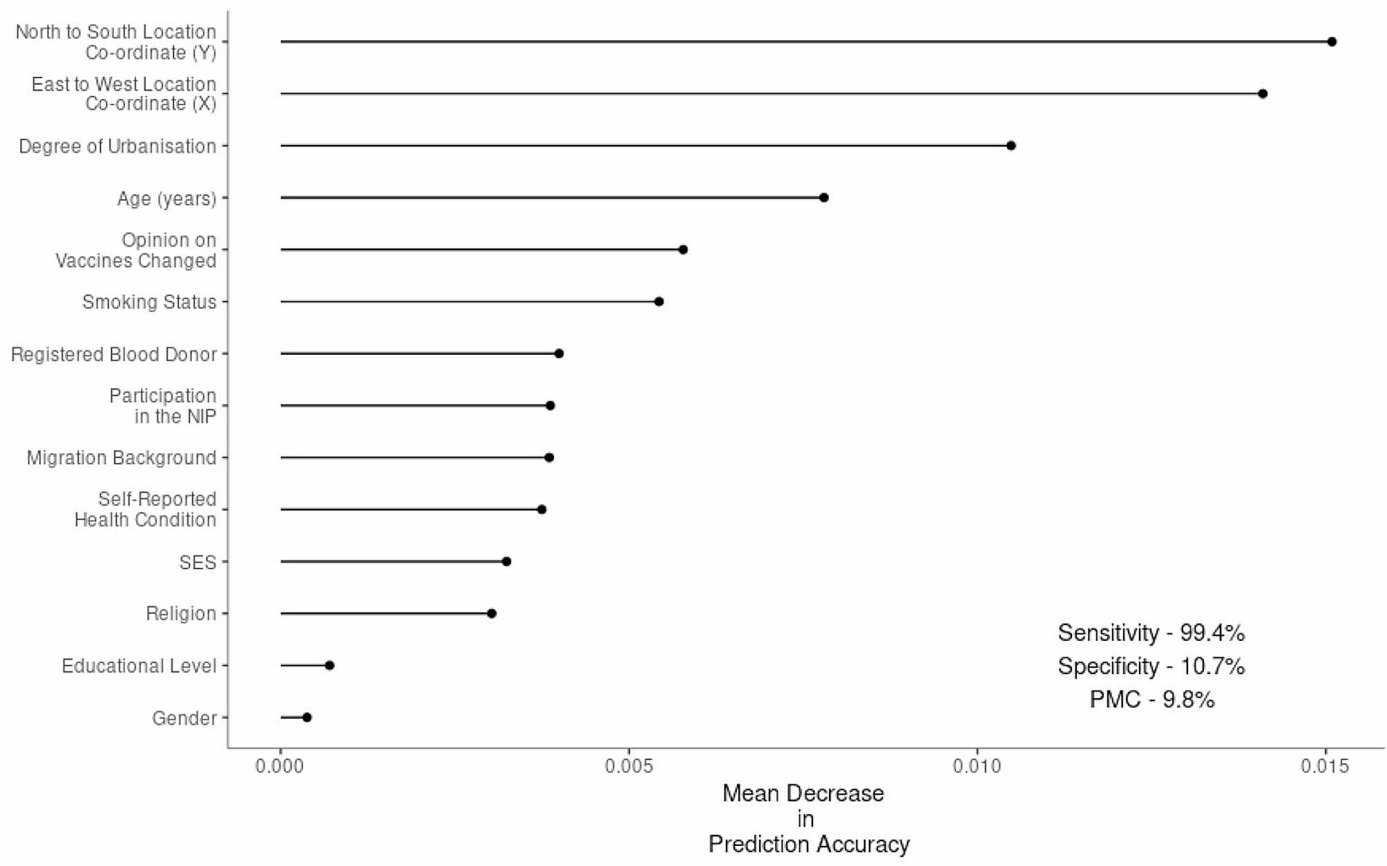
Ordered variable importance for predicting a QO from a FP in P3

When predicting response type among QOs and FPs the most important predictors were geographical location (X and Y co-ordinates) and degree of urbanisation in which the participant lived, closely followed by age (Fig. 4). The model had a pmc of 9.8%, a sensitivity of 59.4%, a specificity of 10.7% and an accuracy (1– pmc) of 90.2% (Table 2). QOs were more likely to reside in very highly urbanised areas and were on average younger than FPs (FP median age 34 (IQR 41), QO median age 29 (IQR 32)) (Table 1). However, due to the distribution of outcome frequency in this comparison (647 QO: 5,553 FP), estimates of sensitivity and specificity were highly unbalanced (Fig. 4) The distribution of QO and FP questionnaire characteristics are provided in Additional File 5.

### Comparison of participants from PIENTER 1 (P1), PIENTER 2 (P2) and PIENTER 3 (P3)

#### Demographics of P1, P2 and P3 full participants (FP)

The overall response rates have decreased considerably between P1 and P3. Non-responders across P1, P2 and P3 mainly alleged lack of time or fear of blood sampling as their reason for non-response (data not shown).

P3 had a lower proportion of children under 9 years than P1 and P2, and there was an increase in the proportion of participants aged 20–29 for each subsequent PIENTER (Table 3). Unsurprisingly, there was a considerable shift from P1 to P3 in the proportion of participants living in high and very highly urbanised areas. Further, there was a large increase in the proportion of participants reporting to have no religious beliefs (Table 3).

**Table 3:** Characteristics of P1, P2 and P3 Full Participants (FPs) from the National Sample only

	PIENTER (Years) Sample Number (Response Rate %)		
	**P1 (1995/1996)** ***N*** ** = 8356 (55.0%)**	**P2 (2006/2007)** ***N*** ** = 5834 (33.9%)**	**P3 (2016/2017)** ***N*** ** = 4897 (15.8%)**
Age group (years)			
0–9	2055 (24.6)	1200 (20.6)	916 (18.7)
10–19	1037 (12.4)	706 (12.1)	561 (11.5)
20–29	719 (8.6)	699 (12.0)	732 (15.0)
30–39	936 (11.2)	699 (12.0)	645 (13.2)
40–49	949 (11.4)	631 (10.8)	573 (11.7)
50–59	996 (11.9)	631 (10.8)	547 (11.2)
60–69	922 (11.0)	721 (12.4)	517 (10.6)
70–79	742 (8.9)	547 (9.4)	406 (8.3)
Sex			
Male	3946 (47.2)	2647 (45.4)	2237 (45.7)
Female	4410 (52.8)	3187 (54.6)	2660 (54.3)
Migration background			
Dutch	7415 (89.0)	4954 (84.9)	4257 (87.0)
Other Western	512 (6.2)	448 (7.7)	342 (7.0)
Moroccan or Turkish	200 (2.4)	153 (2.6)	55 (1.1)
Antillean, Surinamese or Aruban	95 (1.1)	98 (1.7)	56 (1.1)
Other Non-Western	108 (1.3)	181 (3.1)	186 (3.8)
Degree of urbanisation			
Very high	938 (11.2)	834 (14.3)	821 (16.8)
High	1029 (12.3)	1923 (33.0)	1726 (35.3)
Moderate	2166 (25.9)	1281 (22.0)	901 (18.4)
Low	1776 (21.3)	1796 (30.8)	986 (20.1)
Very low	2447 (29.3)	N/A	463 (9.5)
Educational level			
High	1801 (21.6)	1677 (28.8)	1837 (37.5)
Moderate	2437 (29.2)	1922 (32.9)	1632 (33.3)
Low	4008 (48.0)	2181 (37.4)	1138 (23.2)
Missing	110 (1.3)	54 (0.9)	290 (5.9)
Religion			
Protestant	2091 (25.0)	1420 (24.3)	733 (15.0)
Catholic	2801 (33.5)	1692 (29.0)	1086 (22.2)
Other	726 (8.7)	465 (8.0)	314 (6.4)
None	2688 (32.2)	2221 (38.1)	2404 (49.1)
Missing	50 (0.6)	36 (0.6)	360 (7.4)
NIP participation			
Yes/Yes, partly	4988 (59.7)	4037 (69.2)	3687 (75.3)
No	129 (1.5)	111 (1.9)	160 (3.3)
Don’t know	120 (1.4)	153 (2.6)	363 (7.4)
Not eligible	3038 (36.4)	1511 (25.9)	619 (12.6)
Missing	81 (1.0)	22 (0.4)	68 (1.4)
Opinion on vaccination changed			
No	7256 (86.8)	4724 (81.0)	3711 (75.8)
More inclined	760 (9.1)	466 (8.0)	224 (4.6)
Less inclined	154 (1.8)	158 (2.7)	366 (7.5)
Don’t know/ missing	186 (2.2)	486 (8.3)	596 (12.2)
Self-reported Health Condition			
Very good/good	6868 (82.2)	5351 (91.7)	4177 (85.3)
Not good/not bad	929 (11.1)	424 (7.3)	591 (12.1)
Bad/very bad	483 (5.8)	27 (0.5)	59 (1.2)
Don’t know/missing	76 (0.9)	32 (0.6)	70 (1.4)

NIP participation increased with each study, with a concurrent reduction in the proportion of those not eligible for the NIP. Across all three studies, there was an increase in those reporting not to have participated in the NIP despite being eligible. This was accompanied by an increase in those reporting to be less inclined to be vaccinated. Concurrently, there was a reduction in those reporting to have no change in their opinion on vaccination, and a reduction in those reporting to be more inclined to be vaccinated (Table 3).

Table 3. **Characteristics of P1, P2 and P3 Full Participants (FPs) from the National Sample (NS) only (end of manuscript).**

#### Predicting the PIENTER study to which a full participant (FP) belongs

Random forests were used to predict from which PIENTER study a participant originated, from P1 or P2, P2 or P3 and finally from P1 or P3. In all three models the most important variables were participant age and participation in the NIP. Details of the model outputs and variable importance plots are presented in Additional File 6.

## Discussion

As with many large health surveys across Europe, the PIENTER studies face decreasing survey response rates through time [11–13, 26]. With an all-time low response rate to PIENTER3, concerns surrounding the influence of non-response biases on future estimates are at the forefront. However, response rates do not always indicate high levels of non-response bias, and the overall influence of non-response bias on survey-derived estimates varies per research questions [17, 27]. Therefore, the documentation of differences between participants and non-participants is crucial.

### Survey response

The response rates seen in P3 are in line with age and gender stratified response behaviours seen in previous PIENTER studies, and in other large national health surveys [11–13, 26, 28]. Gender imbalances in health survey response are common, and are posited to be mediated by gender-related values interacting with decision making [29, 30]. Despite some research indicating that men may be more likely to respond to a survey when offered higher incentivisation, the larger renumeration offered in P3 has not obviously influenced the gender distribution of the sample [13, 30, 31]. Overall, efforts to increase the numbers of men in the working age ranges in P3 seem to have been largely unsuccessful [13].

Large response differences between the genders in the working age range are commonly seen in other health surveys. In P3 this could largely reflect a perceived burden on time, as this was the most cited reason for non-participation. Further, these differences could be amplified in the Netherlands. In 2017 75% of women aged 20–64 were reported to be working part-time (< 28 h a week), compared to 22% of men [32]. This is more than double the EU28 average for women (31.4%) in this age category [32].

In the non-western migrant (NWM) oversample, the overall response was much lower but varied similarly by age and gender. However, the comparatively high response of Dutch speaking migrants, from Suriname, the Antilles and Aruba (SAN), could indicate a language penetration issue for the survey. The initial invitation and information leaflet were sent in Dutch. There was a single sentence on the second page of the invitation in English, Turkish and Arabic to indicate the letter and information was available in other languages online or at request. These additional steps to access the survey may well reduce individual likelihood of engagement [33]. However, as response was similar between SAN and Other NWMs (non-Dutch speaking), the lower response in those with Turkish or Moroccan backgrounds may reflect additional barriers beyond language. This could relate to variable cultural values surrounding health, research and community engagement, or awareness of and/or trust in the RIVM specifically [34, 35].

### Differentiation of response types

Although random forests were unable to distinguish absolute non-responders (ANRs) from full participants (FPs) accurately, that does not necessarily indicate a lack of non-response bias. In a large meta-analysis of 539 studies, it was demonstrated that prevalence estimates from participants and non-participants often had large differences that were not strikingly evident when comparing the group demographic characteristics [15].

When predicting non-response questionnaire participants (NRQs) from FPs, self-reported health was the strongest predictor, even when coded missingness was excluded in a form of complete-case analysis. FPs reported most frequently to be of very good health, whilst the majority of NRQs, after excluding missing values, reported to be of good health, but not to the higher level seen in FPs. Combined with the large difference in distribution between these health categories in the available data, the high proportion of missing values seen in the NRQs could indicate an unwillingness to divulge poor health status, and thus the presence of healthy responder bias, a well-documented phenomena in voluntary participation health studies [36].

Using the “Continuum of Resistance” theory, which stipulates non-responders are furthest away from full responders on a continuum that ranges from “will never respond” to “will always respond,” we may take the assumption that NRQs act as a reasonable proxy for ANRs. Extending this assumption, we may expect that ANRs on average may report poorer health status. Considerable differences in health status between responders and non-responders to health surveys have been documented previously [28, 37, 38]. As non-response adjustments for demographic factors alone may not reduce estimate biases sufficiently, this could have considerable impacts on health-related and prevalence estimates generated from the P3 sample, depending on the topic [37].

Looking at differentiating FPs from questionnaire only participants (QOs), the most important predictors were related to geographical location, urbanisation, and age. This is probably reflective of perceived available time and survey mode preference, as QOs were younger, and had both a larger proportion of men and those living in areas of very high urbanisation, established predictors of non-response [39].

### Representativeness

We found that the age and gender structure of the P3 sample did not closely mimic that of the Dutch population. This was to be expected due in part to the study design, as sampling was conducted in a stratified manner, with larger numbers of individuals invited in under-5s for example, but also due to differential propensities to participate across age and gender strata. Whilst post-hoc weighting for variables such as age and gender can easily be applied to estimates, adjusting for other factors such as geographical location, urbanisation level, educational level, and health status could prove more difficult. There may well be non-response biases within the weighting classes that are under-represented to begin with, due to the influence of topic saliency, among other factors [15, 18, 27]. Subsequently even those in our sample classified as having lower education and poorer health, for example, may not represent this subgroup well at a population level.

### Participation trends in PIENTER 1, 2, and 3 through the decades

Over the last 30 years the Netherlands has experienced changes in the population structure (e.g., an ageing population), sociological norms, and average educational levels [40–43]. These shifts are somewhat reflected in the numeric comparisons. We saw an increase in the proportion of highly educated participants, a reduction in the proportion of those ascribing to any religious belief system overall and a shift in the distribution of participants across levels of urbanization, with more participants residing in increasingly urban areas over time.

Despite this, PIENTER participant characteristics were not highlighted by the random forests as important in differentiating the studies from each other. In fact, the strongest predictors of study origin were age and NIP participation; in combination a simple proxy for the cohort effect as more of the population becomes eligible for NIP participation, at a younger age, through the years.

However, these differences in PIENTER participant characteristics were not large enough for RF algorithms to differentiate the samples from each other. Based on this we could posit that the PIENTER participants are largely similar “types” of people, and thus estimates from the three studies could be compared across time. This may indicate that it is the interactions between factors, such as the participant characteristics, the social-/physical- environment and the survey topic/mode, that combine to produce a survey participant that have reduced through time [10]. For example, men of working age in highly urbanised areas are least likely to respond to any survey [39]. As the population becomes more urbanised, a larger proportion of this class lives in the city, and so the likelihood of response decreases [40, 44].

Although randomforest was not able to distinguish participants by PIENTER year, we did capture evidence of falling confidence in the NIP among full participants. However, we saw in the variable importance plots that “opinions on vaccination have changed” was of lower but similar importance to “educational level.” It is possible that this apparent falling confidence in the NIP may be a product of the over-representation of the highly educated in the P3 sample. High educational levels have been previously and recently correlated with vaccine hesitancy in Dutch populations [45, 46].

### Limitations

As for all survey research, we faced limitations regarding missing data and data quality. The non-response survey data, conducted as a telephone follow-up, contained a large proportion of item-missing data. Additionally, although the questions in the non-response survey were textually identical to those of the online/paper main survey, the differing modes used to collect the main survey and the non-response survey data may have influenced participants answers. This should be considered alongside our interpretations.

Secondly, our dataset was unbalanced with regard to the response type outcome, and very much so in the QO class. This was indicated by our skewed confusion matrices with low pmcs, and reflected in all of the random forest models sensitivity and specificity values. To check that our conclusions regarding variable importance orders were not distorted by this, we ran analyses on random subsets of data containing more balanced proportions of the two possible outcomes. We found that the rankings of variable importance remained stable. Therefore, whilst these RF algorithms may not generalise well to other datasets, we feel that the description of the most important predictor variables that define response types for PIENTER 3 are valid.

### Future considerations

Adjustments for non-response can only go so far, and it has been shown that a balanced survey response is less biased in its estimates than when using post-hoc adjustments alone [27]. After all, post-hoc weights are frequently based on limited available data, cannot improve overall precision, and do not deal with non-response biases within weighting classes.

However, the primary aim of all three PIENTER studies is to assess the populations seroprevalence of infectious diseases, and levels of protection against vaccine preventable diseases (VPDs). For NIP vaccines in the Netherlands uptake is almost universally high [47]. It is therefore unlikely that any differences between participants and non-participants would have a large impact on estimates of seroprevalence of VPDs. However, this may not be the case when vaccine uptake or disease exposure is less universal, as estimates may become biased where coverage or exposure varies by under-represented subgroups. For example, in the Netherlands, the HPV vaccine was rolled out in 2009 and subsequently included in the NIP during 2010, with uptake reaching a maximum of 63% in 2021 [47, 48]. However, the uptake of the HPV vaccine varies largely by migration background and socioeconomic status [48]. As these groups were under-represented in the PIENTER 3 sample, seroprevalence estimates for HPV vaccines could potentially be biased upwards due to non-coverage of the achieved PIENTER 3 sample.

As survey response is likely to continue to decline, future PIENTER studies may consider alternative study designs and survey methods. For example, the PIENTER 3 sample experienced lower response in working aged men, and non-western migrants. However, a study of survey mode preference in the Netherlands found that those in younger age classes preferred app-based approaches, and that men were more responsive to face-to-face and registration linkage survey methods [44]. Further, it has been demonstrated that additional steps required of an invitee to engage in a survey reduces overall likelihood to participate [10]. As such, future PIENTER surveys might consider targeted mixed-method survey designs to address survey mode preferences across different subgroups. For example, offering an app-based questionnaire and the use of face-to-face recruitment to improve engagement with working-aged groups [49–51], and sending postal questionnaires in multiple languages could act to reduce barriers to participation in non-Dutch speaking communities [10, 34].

## Conclusions

The P3 sample is a powerful and unique tool, adding further biological and epidemiological data to the existing PIENTER biobank. We found that the sample characteristics are broadly the same between the three PIENTER studies, and in combination with comparable study designs this affords the biobank the ability to study trends across 30 years in The Netherlands. Although we found evidence that non-response biases may be present, particularly related to migration background and health, P3 remains a key resource for monitoring population-level protection against VPDs. As vaccination coverage in the Netherlands is generally high, non-response bias due to low coverage may not significantly influence the accuracy of estimates of population seroprevalence of VPDs. However, the power to detect associations between serostatus and behaviours/exposures may be limited, and we urge future researchers using the PIENTER biobank to carefully consider sources of bias on a case-by-case basis.

Unfortunately, as was experienced in PIENTER2, the oversampling of NWMs was not successful across all migration background subgroups. Our findings echo the need for improved coverage of these groups, as previously stated in the non-response analysis of PIENTER2 [52]. Considering this in combination with the continuously decreasing response rates to all surveys, future PIENTER studies may consider alternative sampling and survey methods.

### Electronic supplementary material

Below is the link to the electronic supplementary material.

Supplementary Material 1

Supplementary Material 2

Supplementary Material 3

Supplementary Material 4

Supplementary Material 5

Supplementary Material 6

## Data Availability

The datasets used and/or analysed during the current study are available from the corresponding author on reasonable request. ***Dataset Reference Links***. CBS Population totals 2016 and 2017. https://opendata.cbs.nl/statline/#/CBS/en/dataset/83474ENG/table?dl=6245D. CBS Age, sex, migration background in NL 1st January 2016. https://opendata.cbs.nl/statline/#/CBS/en/dataset/37325eng/table?dl=6245 A. CBS Perceived Health Status in 2013. https://opendata.cbs.nl/statline/#/CBS/en/dataset/81174ENG/table?dl=183FE.

## Abbreviations

NIP: The National Immunisation Programme
PIENTER: Sero–epidemiological study in the Netherlands to monitor the effect of the National Immunization Programme (in Dutch: ‘Peiling Immunisatie Effect Nederland Ter Evaluatie van het Rijksvaccinatieprogramma’)
NWM: Non–Western Migration Background Oversample
DBS: Dried Blood Spot sample
FP: Full Participant
QO: Questionnaire Only participant
NRQ: Non–Response Questionnaire participant
ANR: Absolute Non–Respondent
O: Other response type (excluded)
AAPOR: American Association of Public Health Research
CBS: Central Bureau of Statistics Netherlands
RF: Random forest
Pmc: probability of misclassification
PC4: Postcode at neighbourhood level (first 4 numbers only)
MCAR: missing completely at random
MAR: missing at random
SAN: Suriname, Aruba, and the former Dutch Antilles
RIVM: National Institute for Health and the Environment (in Dutch: ‘Rijksinstituut voor Volksgezondheid en Milieu’)
VPD: Vaccine preventable disease(s)

## References

[1] Giesecke J. Seroepidemiology. Modern infectious disease epidemiology: Hodder Arnold; 2002. p. 188– 98.

[2] Osborne K. JW, E. Miller. The European sero-epidemiology network. Eurosurveillance. 1997;2(4). 10.2807/esm.02.04.00167-en 12631820

[3] Waaijenborg S, Hahne SJ, Mollema L, Smits GP, Berbers GA, van der Klis FR (2013). Waning of maternal antibodies against measles, mumps, rubella, and varicella in communities with contrasting vaccination coverage. J Infect Dis.

[4] Steens A, Mollema L, Berbers GA, van Gageldonk PG, van der Klis FR, de Melker HE (2010). High tetanus antitoxin antibody concentrations in the Netherlands: a seroepidemiological study. Vaccine.

[5] de Voer RM, Mollema L, Schepp RM, de Greeff SC, van Gageldonk PG, de Melker HE (2010). Immunity against Neisseria meningitidis serogroup C in the Dutch population before and after introduction of the meningococcal c conjugate vaccine. PLoS ONE.

[6] Kreuter F (2013). Facing the nonresponse challenge. The ANNALS of the American Academy. Political Social Sci.

[7] Durrant GB, Steele F (2008). Multilevel modelling of Refusal and Non-contact in Household surveys: evidence from six UK Government surveys. J Royal Stat Soc Ser A: Stat Soc.

[8] Roßmann J, Gummer T (2016). Using paradata to Predict and correct for Panel Attrition. Social Sci Comput Rev.

[9] Felderer B, Kueck J, Spindler M. Using double machine learning to Understand Nonresponse in the recruitment of a mixed-Mode Online Panel. Social Sci Comput Rev. 2022.

[10] de Leeuw dH (2002). Trends in Household Survey Nonresponse: a longitudinal and international comparison. Survey Nonresponse.

[11] De Melker HE, Conyn-van Spaendonck MA (1998). Immunosurveillance and the evaluation of national immunization programmes: a population-based approach. Epidemiol Infect.

[12] van der Klis FRM, Berbers LMGAM, de Melker HE, Coutinho RA. Second national serum bank for population-based seroprevalence studies in the Netherlands. Neth J Med. 2009;67(7). 19687529

[13] Verberk JDM, Vos RA, Mollema L, van Vliet J, van Weert JWM, de Melker HE (2019). Third national biobank for population-based seroprevalence studies in the Netherlands, including the Caribbean Netherlands. BMC Infect Dis.

[14] Maitland A, Lin A, Cantor D, Jones M, Moser RP, Hesse BW (2017). A Nonresponse Bias Analysis of the Health Information National Trends Survey (HINTS). J Health Commun.

[15] Groves RM, Peytcheva E (2008). The impact of Nonresponse Rates on Nonresponse Bias: a Meta-analysis. Pub Opin Q.

[16] Ronald C, Kessler RJAL (1995). Groves. Advances in strategies for minimizing and adjusting for Survey Nonresponse. Epidemiol Rev.

[17] Phillips AW, Reddy S, Durning SJ (2016). Improving response rates and evaluating nonresponse bias in surveys: AMEE Guide 102. Med Teach.

[18] Groves RM, Dipko SPS (2004). The role of topic interest in survey participations. Pub Opin Q.

[19] Keyes KM, Rutherford C, Popham F, Martins SS, Gray L (2018). How healthy are Survey respondents compared with the General Population? Using Survey-linked Death records to compare mortality outcomes. Epidemiology.

[20] AAPOR. Standard definitions: final dispositions of Case codes and Outcome Rates for surveys. The American Association of Public Health Research; 2016.

[21] Benjamini Y (1995). Controlling the false Discovery rate: a practical and powerful Approach to multiple testing. J Royal Stat Soc Ser B (Methodological).

[22] Biau G, Scornet E (2016). A random forest guided tour. Test.

[23] Liaw A. MW. Classification and Regression by randomForest. R News - The Newsletter of the R Project. 2002;2(3).

[24] RStudio Team Boston M, RStudio. Integrated Development Environment for R. 2016.

[25] Team RC. R: A language and environment for statistical computing. In: Computing RFfS, editor. Vienna, Austria2019. p. https://www.R-project.org/.

[26] Stedman RC, Connelly NA, Heberlein TA, Decker DJ, Allred SB (2019). The end of the (Research) World as we know it? Understanding and coping with declining response rates to mail surveys. Soc Nat Resour.

[27] Schouten B, Cobben F, Lundquist P, Wagner J (2016). Does more balanced survey response imply lessnon-response bias?. J Royal Stat Soc Ser A.

[28] Tolonen H, Helakorpi S, Talala K, Helasoja V, Martelin T, Prattala R (2006). 25-year trends and socio-demographic differences in response rates: Finnish adult health behaviour survey. Eur J Epidemiol.

[29] Smith WG. Does gender influence online survey participation? A recordlinkage analysis of university faculty online survey response behavior. ERIC Doc Reprod Service. 2008.

[30] Boulianne S (2012). Examining the gender effects of different incentive amounts in a web survey. Field Methods.

[31] Rao N (2020). Cost effectiveness of pre- and post-paid incentives for mail survey response. Surv Pract.

[32] Eurostat. Part-time employment as percentage of the total employment, by sex and age (%) 2016 [Available from: http://appsso.eurostat.ec.europa.eu/nui/show.do?dataset=lfsa_eppga&=en.

[33] Harmsen IA, Bos H, Ruiter RA, Paulussen TG, Kok G, de Melker HE (2015). Vaccination decision-making of immigrant parents in the Netherlands; a focus group study. BMC Public Health.

[34] Ahlmark N, Algren MH, Holmberg T, Norredam ML, Nielsen SS, Blom AB (2015). Survey nonresponse among ethnic minorities in a national health survey–a mixed-method study of participation, barriers, and potentials. Ethn Health.

[35] Slootman M. The Dutch Integration Landscape. Ethnic Identity, Social Mobility and the Role of Soulmates. IMISCOE Research Series2018. p. 59–83.

[36] Manolio TA, Weis BK, Cowie CC, Hoover RN, Hudson K, Kramer BS (2012). New models for large prospective studies: is there a better way?. Am J Epidemiol.

[37] Tolonen H, Laatikainen T, Helakorpi S, Talala K, Martelin T, Prattala R (2010). Marital status, educational level and household income explain part of the excess mortality of survey non-respondents. Eur J Epidemiol.

[38] Torvik FA, Rognmo K, Tambs K (2012). Alcohol use and mental distress as predictors of non-response in a general population health survey: the HUNT study. Soc Psychiatry Psychiatr Epidemiol.

[39] Lynn P (2008). The Problem of Non-response. International Handbook of Survey Methodology.

[40] Centraal Bureau voor de Statistiek (CBS). Nederland in cijfers. 2022.

[41] Centraal Bureau voor. de Statistiek (CBS). Immigratie. 2022.

[42] Centraal Bureau voor de Statistiek (CBS). Voor derde jaar op rij 100 duizend inwoners erbij. 2019.

[43] Pleijers A, de Vries R. Invulling praktisch en theoretisch opgeleiden. In: Centraal Bureau voor de Statistiek (CBS), editor.; 2021.

[44] Mulder J, de Bruijne M (2019). Willingness of online respondents to Participate in Alternative modes of Data Collection. Surv Pract.

[45] Hak E, Schonbeck Y, De Melker H, Van Essen GA, Sanders EA (2005). Negative attitude of highly educated parents and health care workers towards future vaccinations in the Dutch childhood vaccination program. Vaccine.

[46] Veldwijk J, van der Heide I, Rademakers J, Schuit AJ, de Wit GA, Uiters E (2015). Preferences for vaccination: does health literacy make a difference?. Med Decis Mak.

[47] RIVM. The National Immunisation Programme in the Netherlands: Surveillance and Developments 2020–2021. 2021. p. 362.

[48] de Munter AC, Klooster T, van Lier A, Akkermans R, de Melker HE, Ruijs WLM (2021). Determinants of HPV-vaccination uptake and subgroups with a lower uptake in the Netherlands. BMC Public Health.

[49] Christensen AI, Lynn P, Tolstrup JS (2019). Can targeted cover letters improve participation in health surveys? Results from a randomized controlled trial. BMC Med Res Methodol.

[50] Beebe TJ, Jacobson RM, Jenkins SM, Lackore KA, Rutten LJF (2018). Testing the impact of mixed-Mode designs (mail and web) and multiple contact attempts within Mode (Mail or web) on Clinician Survey Response. Health Serv Res.

[51] Lynn P (2017). From standardised to targeted survey procedures for tackling non-response and attrition. Surv Res Methods.

[52] de Melker HE, Nagelkerde NJD (2000). Spaendonck MAEC-v. non-participation in a population-based seroprevalence study of vaccine-preventable diseases. Epidemiol Infect.

