# Supplementary material for: Investigating sources of non-response bias in a population-based seroprevalence study of vaccine-preventable diseases in the Netherlands
Source: BMC Infect Dis. 2024 Feb 23;24:249. doi: 10.1186/s12879-024-09095-5 (PMC10885624; doi:10.1186/s12879-024-09095-5)
Supplement: Supplementary file 1 — Supplementary Material 1 [file 12879_2024_9095_MOESM1_ESM.docx]

**Additional File 1**

Questions included in the shortened non-response follow-up survey. The questions below appear exactly as they appeared in the main questionnaire. An English translation is provided from the third page. Although the questions were the same as in the paper and online survey, the non-response questionnaire was conducted over the telephone by a research call centre.

**1**. Wilt u aangeven wat de belangrijkste reden is waarom u niet heeft deelgenomen aan het PIENTER-onderzoek?

1. Ik zie op tegen bloedafname

2. Geen tijd op aangegeven tijdstip

3. Ik ben/was ziek

4. Ik heb al te veel onderzoeken/prikken gehad

5. Ik ben te oud

6. Anders, namelijk …………………………………………………………………

**2**. Wat is uw geboortedatum?

**3.** U bent:

1. Man

2. Vrouw

**4**. Heeft u meegedaan aan het toen gangbare vaccinatieprogramma/ inentingsprogramma? Deze informatie kunt u misschien in een inentingsboekje opzoeken

1. Ja, alle vaccinaties

2. Ja, maar niet alle vaccinaties

3. Nee

4. Weet ik niet

**5a.** Wat is uw geboorteland?

1. Nederland → ga naar vraag 6 2. Suriname

3. (Voormalige) Nederlandse Antillen

4. Aruba

5. Turkije

6. Marokko

7. Een ander land, namelijk

………………………………….

**5b.** Sinds wanneer woont u in Nederland? Sinds **__** (jaartal)

**6.** Hoe is over het algemeen uw gezondheidstoestand?

1. Zeer goed

2. Goed

3. Gaat wel

4. Slecht

5. Zeer slecht

**7a**. Wat is uw hoogst voltooide opleiding?

1. Geen opleiding (lager onderwijs niet afgemaakt)

2. Lager onderwijs (basisschool, speciaal basisonderwijs)

3. Lager of voorbereidend beroepsonderwijs (zoals LTS, LEAO, LHNO, LBO, VMBO-(BB, KB, GL))

4. Middelbaar algemeen voortgezet onderwijs (zoals MAVO, (M)ULO, MBO-kort, VMBO-TL)

5. Middelbaar beroepsonderwijs en beroepsbegeleidend onderwijs (zoals MBO-lang, MTS, MEAO, BOL, BBL, INAS)

6. Hoger algemeen en voorbereidend wetenschappelijk onderwijs (zoals HAVO, VWO, Atheneum, Gymnasium, HBS, MMS)

7. Hoger beroepsonderwijs (zoals HBO, HTS, HEAO, kandidaatswetenschappelijk onderwijs)

8. Wetenschappelijk onderwijs (universiteit)

**8a**. Tot welk geloof of levensovertuiging rekent u zich?

1. Protestants (Gereformeerd, Hervormd etc.) → ga naar vraag 8b

2. Rooms Katholiek

3. Islam

4. Jodendom

5. Boeddhisme

6. Hindoeïsme

7. Ander geloof of levensovertuiging

8. Geen geloof

**8b**. Tot welk specifiek Protestants kerkgenootschap rekent u zich dan?

1. Protestantse Kerk in Nederland (Nederlands Hervormd, Gereformeerd, Luthers),maar **niet** Gereformeerde Bond

2. Gereformeerde Bond binnen de Protestantse Kerk in Nederland

3. Hersteld Hervormde Kerk

4. Gereformeerde Gemeenten

5. Gereformeerde Gemeenten in Nederland

6. Oud Gereformeerde Gemeenten

7. Christelijke Gereformeerde Kerken

8. Gereformeerde Kerken (vrijgemaakt)

9. Nederlands Gereformeerde Kerken

10. Pinkstergemeenten en Evangeliegemeenten

11. Doopsgezinde Broederschap

12. Remonstrantse Broederschap

13. Baptistengemeenten

14. Anders, namelijk. ................................................

**9.** Heeft één van de onderstaande zaken invloed op uw denkwijze over kindervaccinaties?

1. Antroposofie

2. Homeopathie

3. Natuurgeneeswijzen

4. Geloofsovertuiging

5. Sociale media

6. Anders, namelijk

7. Geen van bovengenoemde antwoorden

**English Translation**

**1**. Please indicate the most important reason as to why you chose NOT to participate in PIENTER research?

1. I am afraid or unwilling to give a blood sample

2. I was unable to attend the allocated study appointment

3. I am/was sick

4. I have already participated in too many research programmes, or I have had too many injections/vaccinations

5. I am too old

6. Other reason(s), for example …………………………………………………………………

**2**. What is your date of birth?

**3.** Are you a:

1. Man

2. Woman

**4**. Have you received vaccinations as part of the national vaccination programme, as was available to you at the time?

1. Yes, all possible vaccinations

2. Yes, but not all vaccinations

3. No

4. I don’t know

**5a.** In what country where you born?

1. The Netherland (is yes, go to question 6) 2. Suriname

3. Former Dutch Antilles

4. Aruba

5. Turkey

6. Morocco

7. Other:

……………………………….

**5b.** In what year did you arrive in The Netherlands?

**6.** In general, how would you rate your health?

1. Very good

2. Good

3. Neither good nor bad

4. Poor

5. Very Poor

**7a**. What is the highest level of education that you have completed?

1. No education

2. Primary school

3. Primary school or vocational preparatory school (for example LTS, LEAO, LHNO, LBO, VMBO-(BB, KB, GL))

4. Secondary school, general education (for example MAVO, (M)ULO, MBO-kort, VMBO-TL)

5. Secondary vocational education and vocational guidance education (for example MBO-lang, MTS, MEAO, BOL, BBL, INAS)

6. Higher secondary general education (for example HAVO, VWO, Atheneum, Gymnasium, HBS, MMS)

7. Higher secondary vocational education (for example HBO, HTS, HEAO, kandidaatswetenschappelijk onderwijs)

8. University study (universiteit)

**8a**. Do you have a faith or belief system?

1. Protestant → Go to question 8b

2. Roman Catholic

3. Islam

4. Judaism

5. Buddhism

6. Hinduism

7. Other belief or faith

8. No belief or faith

**8b**. To which specific Protestant denomination do you belong?

1. Protestant Church in the Netherlands (Dutch Reformed, Reformed, Lutheran), but not Reformed League

2. Reformed League within the Protestant Church in the Netherlands

3. Restored Reformed Church

4. Reformed Churches

5. Reformed Churches in the Netherlands

6. Old Reformed Churches

7. Christian Reformed Churches

8. Reformed Churches (liberated)

9. Dutch Reformed Churches

10. Pentecostalism and Gospel Churches

11. Mennonite Brotherhood

12. Remonstrant Brotherhood

13. Baptist Churches

14. Other, namely. ................................................

**9.** Do any of the following influence your opinions on childhood vaccinations?

1. Anthroposophy

2. Homeopathy

3. Naturopathic medicine

4. Religious beliefs

5. Social media

6. Other… please state

7. None of the these
