# Supplementary material for: Investigating sources of non-response bias in a population-based seroprevalence study of vaccine-preventable diseases in the Netherlands
Source: BMC Infect Dis. 2024 Feb 23;24:249. doi: 10.1186/s12879-024-09095-5 (PMC10885624; doi:10.1186/s12879-024-09095-5)
Supplement: Supplementary file 2 — Supplementary Material 2 [file 12879_2024_9095_MOESM2_ESM.docx]

**Additional File 2- Variable Definitions**

Only municipality register derived variables were used to predict a Response Type (RT) of Absolute Non-Responder (ANR) or Full participant (FP). These included age at time of sampling, gender, migration background, the x and y coordinates of the participant, socio-economic status (SES) and degree of urbanization.

Additional data from the non-response questionnaire was combined with the municipality registration data mentioned above to predict a RT of Non-Response Questionnaire (NRQ) or FP. Additional variables included self-reported health satisfaction, previous participation in the Dutch NIP and religious belief.

All of the above variables were combined with additional questionnaire derived variables to predict a RT of Questionnaire Only (QO) or FP. These included educational level, smoking status, and changed opinion on vaccination. All variable definitions are giving below. There were two versions of the questionnaire, for children aged 0-14 years and for those ages 15 and older. The questions were presented in the same manner across both questionnaires, however parents/guardians completed the 0-14 questionnaire on behalf of the child.

**Migration Background**

Migration background was taken directly from municipality registration records. Migration background definitions were derived from Statistics Netherlands (CBS). The participants place of birth and the place of birth of both or either parents were considered.

Categories were:

- Dutch - Person of whom both parents were born in the Netherlands, regardless of the country where the person was born
- Non-Western Migrant - Person with a migration background from one of the countries in Africa, Latin America and Asia (excluding Indonesia and Japan) or Turkey.
- Western Migrant - Person with migration background from one of the countries in Europe (excluding Turkey), North America and Oceania, and Indonesia and Japan.

Categories were further divided into 1^st^ and 2^nd^ generation. A person with a first-generation migration background has the country where he or she was born as the migration background. A person with a second-generation migration background has the mother's country of birth as the migration background, unless that is also the Netherlands. In that case, the migration background is determined by the father's country of birth.

For the purpose of this manuscript, 1^st^ and 2^nd^ generation were grouped together. Non-western migrants were stratified into people with migration backgrounds in “Morocco and Turkey”, “Suriname, Aruba or the Antilles” and “All other non-western”.

**https://www.cbs.nl/nl-nl/onze-diensten/methoden/begrippen/migratieachtergrond**

**Socioeconomic Status (SES)**

SES was generated from 2016 status scores calculated by the Sociaal en Cultureel Planbureau (SCP). Status scores are compound measures that consider the average income, the percentage of people with a low income, the percentage of people with low educational levels and the percentage of unemployment (PC4 level). For this analysis the scores were ranked and then divided into quintiles (1 – low SES to 5 – high SES) before being matched to P3 invitees based on PC4 level postcode. Where a 2016 score was not available, a 2017 score was used.

**Degree of Urbanisation**

The degree of urbanisation was based on the population density within a 1km radius of the invitees address. Definitions were matched to those of the CBS, and were grouped into five categories based on population density.

- 1– Very heavily urbanised (> 2000 persons per km^2^)
- 2– Heavily urbanised (1000 to 2000 pp km^2^)
- 3– Medium urbanisation (500 to 1000 pp km^2^)
- 4– Low urbanisation (250 to 500 pp km^2^)
- 5– No urbanisation/Rural (<250 pp km^2^)).

**X and Y Co-ordinates**

X and Y co-ordinates were generated using ArcGIS, using the neighbourhood level postcode of the invitee (PC4, the first 4 letters of the postcode, “wijk” level). PC4 was obtained from the municipality record. The use of postcode locations gives more granularity than using municipality level clusters, and may indicate variations in socioeconomic status by neighbourhood level. Co-ordinates were used in place of the postcode as random forest performs better with numeric variables in place of factor variables with large numbers of factor levels.

**Questionnaire Derived Variables**

**Self-Reported Health Satisfaction**

“How satisfied are you with your health?”

Categorical, 6 levels; very good, good, fine, bad, very bad and missing.

The grouping of this variable was not adjusted for the analysis.

**NIP Participation**

“As a child did you participate in the NIP at the time/have you ever participated in the immunisation programme?”

Categorical, 5 levels; yes to all or some vaccinations, never participated, don’t know, not eligible and missing.

For the analysis, two levels were grouped together. In the questionnaire(s), “Yes to all vaccinations” and “Yes to some vaccinations” were discrete options. We grouped these participants together as the number reporting “Yes to some vaccinations” was very small.

**Religious beliefs**

“To which religion or belief system do you belong?”

Categorical, 4 levels; Protestant, Other religion or belief system, no faith, missing

Groups were adjusted for the analysis. In the Netherlands orthodox protestants are a group of interest, as they are geographically clustered and have lower vaccination uptake than the Netherlands average. As such, we used the groups: Protestant, Other Religious Belief, No Faith and Missing. Other religious belief included: Roman Catholic, Islam, Judaism, Buddhism, Hinduism, other religion or belief system.

**Educational Level**

“What is your highest level of completed education?”

1. No education (primary education not completed)

2. Primary education (primary school, special primary education)

3. Primary or preparatory vocational education (such as LTS, LEAO, LHNO, LBO, VMBO (BB, KB, GL)

4. Secondary general secondary education (such as MAVO, (M) ULO, MBO-short, VMBO-TL)

5.Secondary vocational education and vocational guidance (such as MBO-long, MTS, MEAO, BOL, BBL, INAS)

6. Higher general and preparatory scientific education (such as HAVO, VWO, Atheneum, Gymnasium, HBS, MMS)

7. Higher vocational education (asHBO, HTS, HEAO, candidate-science education)

8. University education

Categorical, 4 levels; low, middle, high, missing.

For the analysis, low education comprised of option 1 to 4; middle option 5 to 6; high option 7 and 8. For children under 15 years of age, the highest educational level achieved by either parent/guardian was used.

**Smoking Status**

“Do you (sometimes) smoke? If yes, how much do you smoke?”

- Yes;
  - About ___ cigarettes per week
  - About ___ cigars per week
  - About ___ pack(s) of pipe tobacco per week
  - About ___ e-cigarettes per week
  - About ___ time a week smoking a waterpipe
- No, I used to smoke but I quit ___ years ago
- No, I’ve never smoked

Categorical, 3 levels; yes, no, missing

All responses of yes assigned to “yes”, irrespective of smoking frequency or type. Former smokers and never smokers were all assigned to “no”.

**Opinion of vaccination changed**

“Has your opinion about vaccination changed in the past 5 years?”

Categorical, 5 levels; Yes more inclined to vaccinate, Yes less inclined to vaccinate, No, I don’t know, missing.
