# Supplementary material for: Investigating sources of non-response bias in a population-based seroprevalence study of vaccine-preventable diseases in the Netherlands
Source: BMC Infect Dis. 2024 Feb 23;24:249. doi: 10.1186/s12879-024-09095-5 (PMC10885624; doi:10.1186/s12879-024-09095-5)
Supplement: Supplementary file 3 — Supplementary Material 3 [file 12879_2024_9095_MOESM3_ESM.docx]

**Additional File 3**

**Figure S1.** Panel A- American Association of Population Outcomes Research (AAPOR) 2016 Response Rate 1 by age-category and gender for the National Sample; Panel B - AAPOR 2016 Response Rate 1 by age-category and gender for the Non-Western Migrant Sample


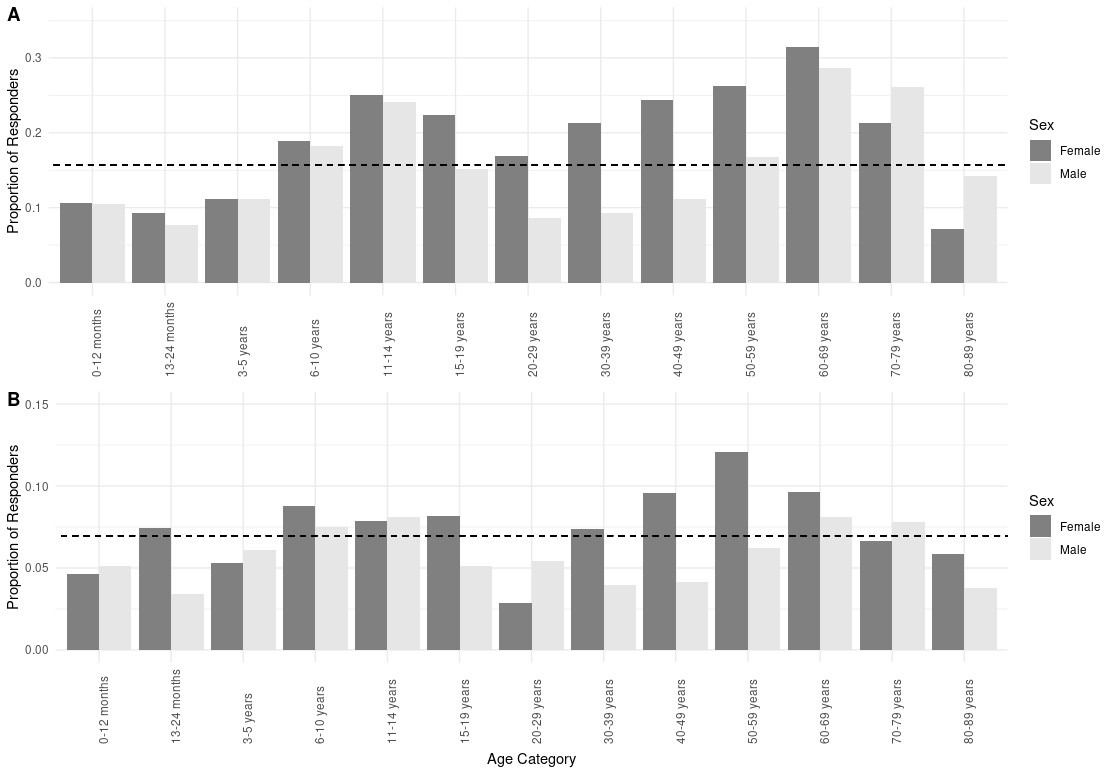


**
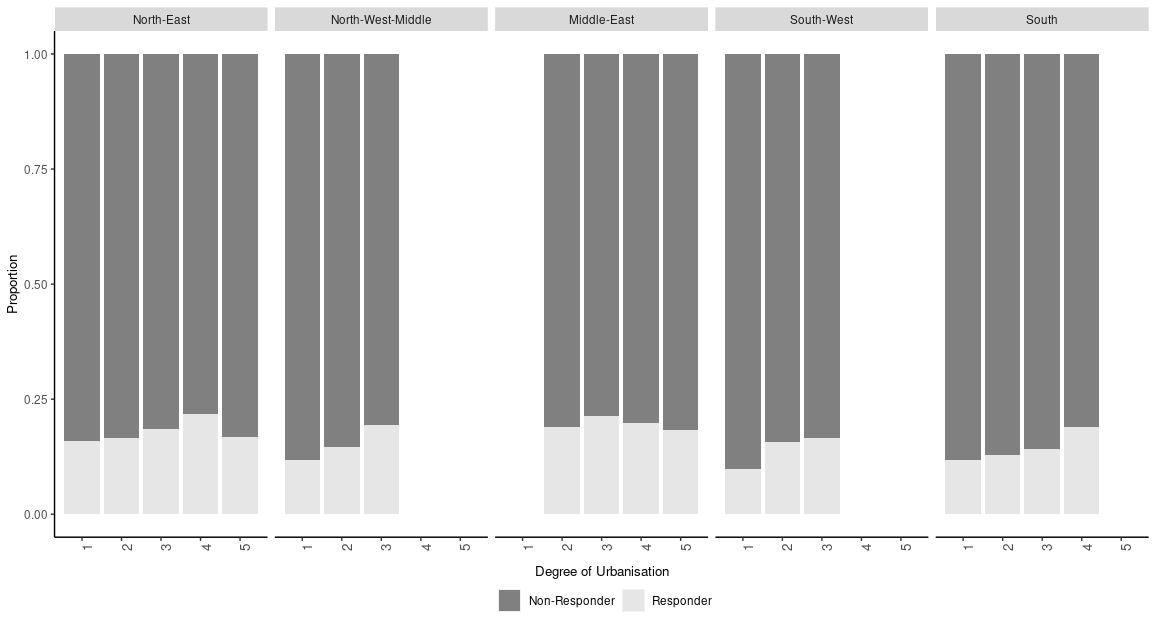
Figure S2.** AAPOR 2016 Response Rate 1 in each of the 5 regions, by degree of urbanisation. Denoted as; 1– Very heavily urbanised (> 2000 persons per km^2^) , 2– Heavily urbanised (1000 to 2000 pp km^2^), 3– Medium urbanisation (500 to 1000 pp km^2^), 4– Low urbanisation (250 to 500 pp km^2^) , 5– No urbanisation/Rural (<250 pp km^2^).
