# Supplementary material for: Investigating sources of non-response bias in a population-based seroprevalence study of vaccine-preventable diseases in the Netherlands
Source: BMC Infect Dis. 2024 Feb 23;24:249. doi: 10.1186/s12879-024-09095-5 (PMC10885624; doi:10.1186/s12879-024-09095-5)
Supplement: Supplementary file 4 — Supplementary Material 4 [file 12879_2024_9095_MOESM4_ESM.docx]

|  |  | **PIENTER3**  **Full Participants** | | **Dutch Population (2016)** | |
| --- | --- | --- | --- | --- | --- |
|  |  | **N** | **%** | **N** | **%** |
| **Sex** | Total | 5,553 | --- | 16,859,609 | --- |
|  | Male | 2,538 | 45.7 | 8,386,302 | 49.7 |
|  | Female | 3,015 | 54.3 | 8,473,306 | 50.3 |
| **Age at** | <10 | 1,051 | 18.9 | 1,806,892 | 10.7 |
| **Recruitment** | 10-19 | 608 | 10.9 | 2,011,607 | 11.9 |
|  | 20-29 | 766 | 13.8 | 2,136,948 | 12.7 |
|  | 30-39 | 697 | 12.6 | 2,026,754 | 12.0 |
|  | 40-49 | 634 | 11.4 | 2,406,726 | 14.3 |
|  | 50-59 | 613 | 11.0 | 2,451,133 | 14.5 |
|  | 60-69 | 643 | 11.6 | 2,100,455 | 12.5 |
|  | 70-79 | 447 | 8.0 | 1,289,857 | 7.7 |
|  | 80+ | 94 | 1.7 | 629,236 | 3.7 |
| **Migration** | Dutch | 4,352 | 78.4 | 13,226,829 | 77.90 |
| **Background** | Other Western | 366 | 6.6 | 1,655,699 | 9.8 |
|  | Morocco or Turkey | 134 | 2.4 | 783,232 | 4.6 |
|  | Suriname, Antilles or Aruba | 269 | 4.8 | 500,003 | 2.9 |
|  | Other Non-Western | 431 | 7.8 | 813,357 | 4.8 |
|  | Missing | 1 | 0.02 | --- | --- |
| **Region** | North-East | 1,100 | 19.8 | 2,862,670 | 16.9 |
|  | North-West | 932 | 16.8 | 3,308,964 | 19.5 |
|  | Central | 1,054 | 19.0 | 3,188,922 | 18.8 |
|  | South-West | 1,058 | 19.1 | 4,003,555 | 23.6 |
|  | South-East | 1,409 | 25.4 | 3,615,009 | 21.3 |
| **Degree of** | Very high | 1,167 | 21.0 | 3,905,490 | 23.0 |
| **Urbanization** | High | 1,816 | 32.7 | 4,218,960 | 24.8 |
|  | Middle | 1,064 | 19.2 | 2,944,460 | 17.3 |
|  | Low | 1,018 | 18.3 | 2,953,260 | 17.4 |
|  | Very low | 488 | 8.8 | 2,956,950 | 17.4 |
| **Education for >15 Years** | Low | 1,198 | 28.8 | 4,522,000 | 32.3 |
|  | Middle | 1,336 | 32.1 | 5,268,000 | 37.7 |
|  | High | 1,393 | 33.5 | 4,000,000 | 28.6 |
|  | Missing | 237 | 5.7 | --- | --- |
| **Income** | Low | 682 | 12.3 | 2,472,500 | 32.4 |
|  | Middle | 2,170 | 39.1 | 4,029,800 | 52.9 |
|  | High | 1,869 | 33.7 | 1,120,900 | 14.7 |
|  | Missing | 832 | 15.0 | --- | --- |
| **Religion** | No religion | 2,525 | 45.5 | NR | 49.0 |
|  | Roman Catholic | 1,231 | 22.2 | NR | 25.0 |
|  | Protestant | 826 | 14.9 | NR | 16.0 |
|  | Other | 580 | 10.4 | NR | 10.0 |
|  | Missing | 391 | 7.0 | --- | --- |
| **Blood donors*** | Registered Blood Donor  (18 – 64 years) | 588 | 18.8 | 330,000* | 2.50 |
| **Self-Reported** | Dissatisfied | 99 | 1.78 | NR | 9.90 |
| **Health Satisfaction** | Neither | 752 | 13.5 | NR | 20.70 |
|  | Satisfied | 4,620 | 83.2 | NR | 69.40 |
|  | Missing | 82 | 1.5 | --- | --- |
|  | | | | | |
| NR – not reported \| --- Not Applicable \| *As reported by Sanquin available at: <https://www.sanquin.org/research/donor-insight/measurements/dutch-donor-database> (accessed 11 June 2019) | | | | | |

**Additional File 4**

**Table S1. Characteristics of PIENTER 3 Full Participants in Comparison to the Dutch National Population Statistics Netherlands**
