# Supplementary material for: Investigating sources of non-response bias in a population-based seroprevalence study of vaccine-preventable diseases in the Netherlands
Source: BMC Infect Dis. 2024 Feb 23;24:249. doi: 10.1186/s12879-024-09095-5 (PMC10885624; doi:10.1186/s12879-024-09095-5)
Supplement: Supplementary file 5 — Supplementary Material 5 [file 12879_2024_9095_MOESM5_ESM.docx]

| Variable | Full Participants  (FPs)  N = 5,553 | Questionnaire Only Participants (QOs)  N = 647 | Non-Response Questionnaire Participants (NRQs)  N =14,043 |
| --- | --- | --- | --- |
| Health Satisfaction | **n (%)** | **n (%)** | **n (%)** |
| Very good | 1434 (25.8) | 145 (22.4) | 707 (5.0) |
| Good | 3250 (58.5) | 347 (53.6) | 7270 (51.8) |
| Fine | 704 (12.7) | 70 (10.8) | 585 (4.2) |
| Bad | 76 (1.4) | 5 (0.8) | 113 (0.8) |
| Very Bad | 12 (0.2) | 3 (0.5) | 12 (0.1) |
| Missing | 77 (1.4) | 77 (11.9) | 5356 (38.1) |
| Religion | **n (%)** | **n (%)** | **n (%)** |
| Protestant | 827 (14.9) | 61 (9.4) | 585 (4.2) |
| Catholic | 1244 (22.4) | 136 (21.0) | 1684 (12.0) |
| Other | 581 (10.5) | 71 (11.0) | 605 (4.3) |
| None | 2559 (46.1) | 310 (47.9) | 5632 (40.1) |
| Missing | 342 (6.2) | 69 (10.7) | 5537 (39.4) |
| NIP participation | **n (%)** | **n (%)** | **n (%)** |
| Fully or partly | 4004 (72.1) | 429 (66.3) | 7946 (56.6) |
| No | 239 (4.3) | 36 (5.6) | 517 (3.7) |
| Don’t know | 439 (7.9) | 73 (11.3) | 341 (2.4) |
| Not eligible | 791 (14.2) | 53 (8.2) | 601 (4.3) |
| Missing | 80 (1.4) | 56 (8.7) | 4639 (33.0) |
| Education | **n (%)** | **n (%)** | **n (%)** |
| Low | 1330 (24.0) | 134 (20.7) | 1869 (13.3) |
| Middle | 1690 (30.4) | 199 (30.8) | 391 (2.8) |
| High | 2176 (39.2) | 238 (36.8) | 120 (0.8) |
| Missing | 357 (6.4) | 76 (11.7) | 11663 (83.1) |
| Opinion vaccination changed | **n (%)** | **n (%)** | **n (%)** |
| Yes, more inclined | 265 (4.8) | 23 (3.6) | -- |
| Yes, less inclined | 398 (7.2) | 40 (6.2) | -- |
| No | 4097 (73.8) | 428 (66.2) | -- |
| Don’t know | 472 (8.5) | 48 (7.4) | -- |
| Missing | 321 (5.8) | 108 (16.7) | -- |
| Smoking Status | **n (%)** | **n (%)** | **n(%)** |
| Current Smoker | 584 (10.5) | 69 (10.7) | -- |
| Former Smoker | 1265 (22.8) | 113 (17.5) | -- |
| Never Smoked | 3179 (57.2) | 311 (48.1) | -- |
| Missing | 525 (9.5) | 154 (23.8) | -- |

**Additional File 5**

**Table S2.** Distribution of counts and percentages of participants by response type, for variables included in the Random Forest (RF) analyses.

All variables for the Absolute Non-Responder (ANR) analysis are described in Table 2 of the manuscript.

| **Variables** | **FPs** | | **NRQs** | |
| --- | --- | --- | --- | --- |
|  | **N = 5546*** | | **N = 9451*** | |
| **Health Satisfaction** | **n (%)** | | **n (%)** | |
| Very good | 1434 | (25.9%) | 707 | (7.5%) |
| Good | 3250 | (58.6%) | 7270 | (76.9%) |
| Fine | 704 | (12.7%) | 585 | (6.2%) |
| Bad | 76 | (1.4%) | 113 | (1.2%) |
| Very Bad | 12 | (0.2%) | 12 | (0.1%) |
| Missing | 70 | (1.3%) | 764 | (8.1%) |
| **Religion** |  |  |  |  |
| Protestant | 826 | (14.9%) | 585 | (6.2%) |
| Catholic | 1231 | (22.2%) | 1684 | (17.8%) |
| Other | 292 | (5.3%) | 605 | (6.4%) |
| None | 2525 | (45.5%) | 5632 | (59.6%) |
| Missing | 384 | (6.9%) | 1040 | (11.0%) |
| **NIP participation** |  |  |  |  |
| Fully or partly | 4424 | (79.8%) | 7946 | (84.1%) |
| No | 353 | (6.4%) | 517 | (5.5%) |
| Don’t know | 673 | (12.1%) | 341 | (3.6%) |
| Not eligible | 23 | (0.4%) | 600 | (6.3%) |
| Missing | 73 | (1.3%) | 47 | (0.5%) |

**Table S3.** Distribution of counts and percentages of non-response questionnaire participants (NRQs) and full participants (FPs) after excluding participants missing data for all three NRQ variables ; health satisfaction, religion, NIP participation

*excluding those with missing data for all three NRQ variables
