# Supplementary material for: Investigating sources of non-response bias in a population-based seroprevalence study of vaccine-preventable diseases in the Netherlands
Source: BMC Infect Dis. 2024 Feb 23;24:249. doi: 10.1186/s12879-024-09095-5 (PMC10885624; doi:10.1186/s12879-024-09095-5)
Supplement: Supplementary file 6 — Supplementary Material 6 [file 12879_2024_9095_MOESM6_ESM.docx]

**Additional File 6**

**Table S4.** Results of Random Forest predicting PIENTER study of origin for National Sample FPs

| **Model*** | **Sensitivity (%)** | **Specificity (%)** | **PMC (%)** | **Most important variables** |
| --- | --- | --- | --- | --- |
| **P1 (0) or P2 (1)** | 84.3 | 43.4 | 33.0 | Age;  Participation in the NIP |
| **P2 (0) or P3 (1)** | 79.4 | 52.2 | 33.0 | Age;  Religion;  Participation in the NIP |
| **P1 (0) or P3 (1)** | 87.9 | 66.3 | 20.0 | Age;  Participation in the NIP |

* 0 indicates the negative outcome, 1 indicates the positive outcome

**
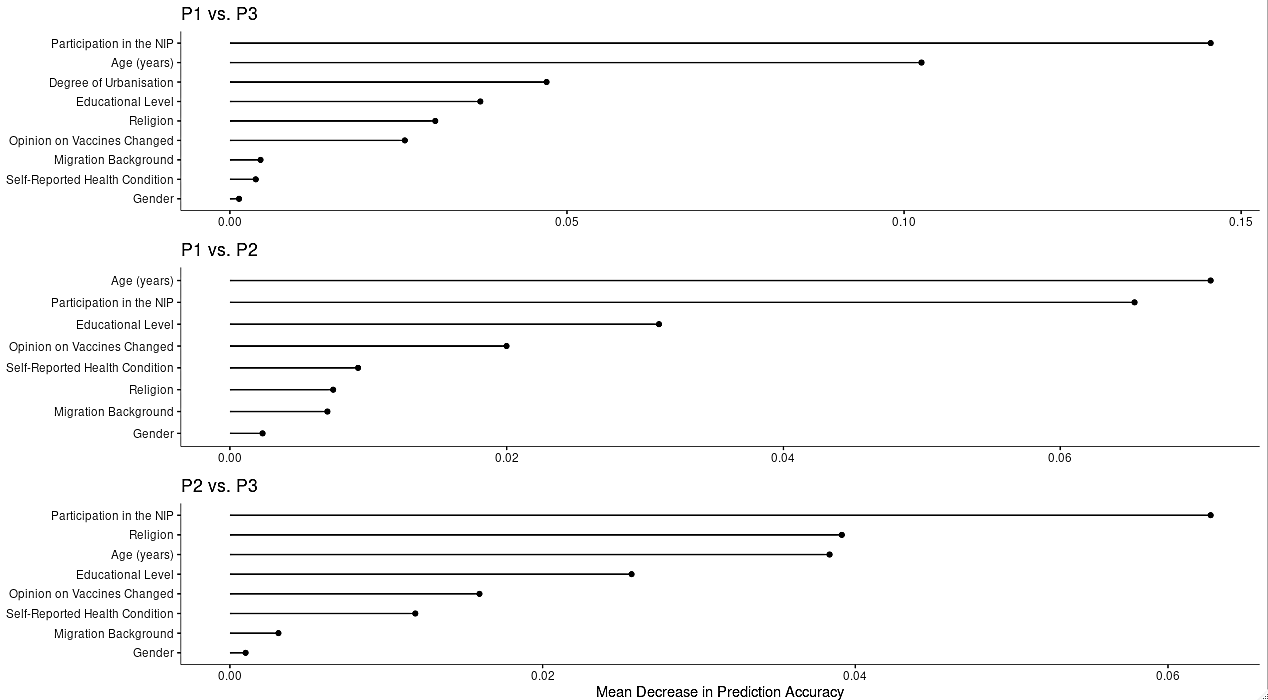
Figure S3.** Ordered variable importance for prediction of PIENTER Study Participation.
